# *SMALL PLANT* AND *ORGAN 1* (*SPO1*) Encoding a Cellulose Synthase-like Protein D4 (OsCSLD4) Is an Important Regulator for Plant Architecture and Organ Size in Rice

**DOI:** 10.3390/ijms242316974

**Published:** 2023-11-30

**Authors:** Lei Qiao, Qilong Wu, Liuzhen Yuan, Xudong Huang, Yutao Yang, Qinying Li, Nida Shahzad, Haifeng Li, Wenqiang Li

**Affiliations:** 1 State Key Laboratory of Crop Stress Biology in Arid Areas, College of Life Sciences, Northwest A&F University, Yangling 712100, Chinahxd@nwafu.edu.cn (X.H.); yangyutao224@163.com (Y.Y.); li15725480698@163.com (Q.L.); nidashahzad811@gmail.com (N.S.); 2 College of Agronomy, Northwest A&F University, Yangling 712100, China

**Keywords:** *SPO1*/*OsCSLD4*, narrow and rolled leaf, plant architecture, organ size, plant hormone, cell division and expansion

## Abstract

Plant architecture and organ size are considered as important traits in crop breeding and germplasm improvement. Although several factors affecting plant architecture and organ size have been identified in rice, the genetic and regulatory mechanisms remain to be elucidated. Here, we identified and characterized the *small plant and organ 1* (*spo1*) mutant in rice (*Oryza sativa*), which exhibits narrow and rolled leaf, reductions in plant height, root length, and grain width, and other morphological defects. Map-based cloning revealed that *SPO1* is allelic with *OsCSLD4*, a gene encoding the cellulose synthase-like protein D4, and is highly expressed in the roots at the seedling and tillering stages. Microscopic observation revealed the *spo1* mutant had reduced number and width in leaf veins, smaller size of leaf bulliform cells, reduced cell length and cell area in the culm, and decreased width of epidermal cells in the outer glume of the grain. These results indicate the role of *SPO1* in modulating cell division and cell expansion, which modulates plant architecture and organ size. It is showed that the contents of endogenous hormones including auxin, abscisic acid, gibberellin, and zeatin tested in the *spo1* mutant were significantly altered, compared to the wild type. Furthermore, the transcriptome analysis revealed that the differentially expressed genes (DEGs) are significantly enriched in the pathways associated with plant hormone signal transduction, cell cycle progression, and cell wall formation. These results indicated that the loss of *SPO1*/*OsCSLD4* function disrupted cell wall cellulose synthase and hormones homeostasis and signaling, thus leading to smaller plant and organ size in *spo1*. Taken together, we suggest the functional role of *SPO1*/*OsCSLD4* in the control of rice plant and organ size by modulating cell division and expansion, likely through the effects of multiple hormonal pathways on cell wall formation.

## 1. Introduction

Rice (*Oryza sativa* L.) is one of the most important food crops in the world. Increasing plant biomass, especially grain yield, has always been a primary goal of rice breeding and germplasm improvement [1]. Plant architecture including leaf morphology, plant height, tiller patterning, and reproductive organ structure, etc., is closely associated with rice cultivation, harvest index, biomass, and grain yield, being of great agronomic importance [2,3]. At the cellular level, these agronomic traits, such as leaf shape, plant height, and grain size, also known as organ size, are determined by cell number and cell size, resulting from the processes of cell proliferation and cell expansion [4,5]. Therefore, elucidation of the genetic and molecular mechanisms controlling plant architecture and organ size may contribute to rice breeding and improvement.

The leaf is the main photosynthetic and gas-exchange organ in rice. Leaf shape affects energy capture and other important physiological activities that have a close connection with plant architecture and grain yield [6,7]. So far, a large number of genes have been reported to regulate leaf shape, particularly leaf width and leaf rolling. A series of characterized genes in rice, such as *NAL1* [8], *NAL2/3* [9], *NAL9* [10], *NAL11* [11], *NRL2* [12], *DNL-4* [13], *NAL21* [14], *WL1* [15], and *NAL22* [16], control leaf width mainly by regulating the number of leaf veins, the distance between veins, and the width of veins. Rice leaf rolling is often associated with the development of bulliform cells (BCs) in the adaxial side of the leaves. Changes in the size and/or number of BCs may cause varying degrees of leaf rolling, such as *NAL7* [17], *SLL1* [18], *ACL1* and *ACL2* [19], *ROC5* [20], *SRL1* [21], *RL14* [22], *OsZHD1* [23], *OsHox32* [24], and *REL2* [25]. Moreover, some genes not only regulate leaf shape but also play a role in determining plant height and/or grain size, such as *NRL2* [12], *DNL-4* [13], *NAL21* [14], *REL2* [25], *NAL1* [26], *miR159* [27] *AH2*/*SLL1* [28], and *CLD*/*SRL1* [29]. It is suggested that leaf shape, plant height, and grain size are affected by the number and/or size of cells. For example, the *nal1* exhibits narrow leaves due to defects in anticlinal cell division and reduced abaxial epidermal cell size [26,30]. The semi-dwarf mutant *cpb1* is characterized by shorter stem internodes, mainly resulting from shorter cell length [31]. Rice *OsCBL5* promotes grain size by affecting the expansion of spikelet hull cells [32]. Rice *GS9* regulates grain shape by affecting the number of cells in the spikelet hull [33]. Therefore, genes regulating cell division and/or expansion play important roles in determining plant architecture and organ size, which are determined by cell number and/or size. Furthermore, in plants, hormones can also play critical roles in plant architecture and organ size regulation. Many hormone-related genes have been characterized as important regulators of plant architecture and organ size in rice [34,35]. Among the hormones, gibberellins (GAs) and auxin (IAA) are mainly responsible for cell division and expansion in controlling organ size [36,37]. Blocked GA synthesis and signaling usually show reduced height and small grain [36,38]. Auxin deficiency and/or insensitivity may alter plant height, leaf width, and grain size [39,40]. Rice plants overexpressing *OsGA2ox5*, a gene encoding gibberellin (GA) 2-oxidases, exhibited dominant dwarf and GA-deficient phenotypes, with shorter stems resulting in shorter and smaller cells of leaf sheaths and later development of reproductive organs [41]. *GW6* (*GRAIN WIDTH 6*) encodes a GA-regulated GAST family protein and positively regulates grain width by promoting cell expansion in the spikelet hull and by modulating GA response and biosynthesis in rice [42]. The *OsARF11* gene contributes to the reduced growth of roots and leaf blades with a reduced response to exogenous auxin, which is consistent with its role in mediating a response to auxin to stimulate cell division and/or cell expansion [43]. Activation of *BIG GRAIN1* (*BG1*), a rice gene regulating auxin transport, results in large grains due to increased cell proliferation and cell expansion in spikelet hulls [44]. Furthermore, some hormones may play overlapping roles and have crosstalk in regulating plant architecture and organ size [45,46,47,48]. It is suggested that *OsSHI1* can act as a transcriptional regulatory hub that orchestrates the integration and self-feedback regulation of multiple hormone signaling pathways including auxin, BRs, and ABA to coordinate plant architecture and other aspects [49]. *OsIAA1*-overexpressing transgenic plants have decreased plant height and loose plant architecture, followed by reduced auxin sensitivity but increased sensitivity to BR [50]. These studies suggest that the sophisticated regulatory mechanism coordinates plant architecture and organ size by integrating various plant hormone signaling pathways to regulate cell division and/or cell expansion in rice.

Plant cell walls mainly comprise cellulose, hemicellulose, pectin, and structural proteins [51]. Cellulose is produced in the plasma membrane by cellulose synthase complexes, and cellulose synthase-like (CSL) superfamily genes play a very important role in this process [52]. Besides the functions in regulation of cell wall and cellulose biosynthesis, CSL members are also involved in other aspects of plant growth and development, such as the roles of maize *CSLD1* [53] and Arabidopsis *SOS6*/*AtCSLD5* [54] in cell division, expansion, and abiotic stress response, etc. As one of the five *CSLD* genes in rice, the *OsCSLD4* gene has been extensively studied in recent years [55,56,57,58,59,60,61,62,63,64]. Although the different mutant alleles display more or less difference in plant phenotype, a common characteristic in *OsCSLD4* gene mutation is the defects in plant and leaf morphology. For instance, the *dnl1* mutant displayed dwarf and narrowed phenotype [60], the *nrl1* mutant showed fewer veins and smaller adaxial bulliform cells in leaf blade and decreased in plant height [56], and the *nrl1* mutant showed a decrease in the number of vascular bundles of leaf [57]. It was revealed that the loss of *OsCSLD4* gene function altered the structure of arabinoxylan and the content of cellulose and homogalacturonan in *nd1* mutant [55] and altered the cellulose content and the level of xylose in *cd1* mutant [58]. Yoshikawa et al. (2013) [59] suggested that the product of *OsCSLD4* plays a pivotal role in the M phase to regulate cell proliferation. Shi et al. (2016) [61] indicated the role of *OsCSLD4* in gibberellin signaling by characterization of the *dnl3* mutant. Furthermore, it demonstrated the function of *OSCSLD4* in the regulation of abiotic stresses including salt, salt–alkali, and drought stress [62,63,64]. These results indicate that *OsCSLD4* is an important regulator in plant growth and development, abiotic stress response, and other aspects.

Although genes regulating plant architecture and organ size have been extensively identified in rice, the underlying regulatory mechanisms are still under investigation. In this study, we identified and characterized *small plant and organ 1* (*spo1*), a new mutant allele of *OsCSLD4*, which showed narrow and rolled leaves, decreased plant height, root length, and grain width, and other morphological defects. Our investigation revealed that the loss-of-function of *SPO1*/*OsCSLD4* led to a reduced number and width in leaf veins, smaller size of leaf bulliform cells, reduced cell length and cell area in the culm, and decreased width of epidermal cells in the grain, and was accompanied with significant changes in multiple hormone contents and altered expressions of genes associated with plant hormone signal transduction, cell cycle progression, and cell wall formation. Considering that *SPO1*/*OsCSLD4* encoding cellulose synthase-like D4 protein is required for cell wall cellulose formation, these results suggested that *SPO1*/*OsCSLD4* plays an important role in regulating plant and organ size through cell division and expansion, possibly mediated by the effects of multiple hormone pathways on cell wall formation.

## 2. Results

### 2.1. Phenotypic Characterization of the spo1 Mutant

**Figure 1:**
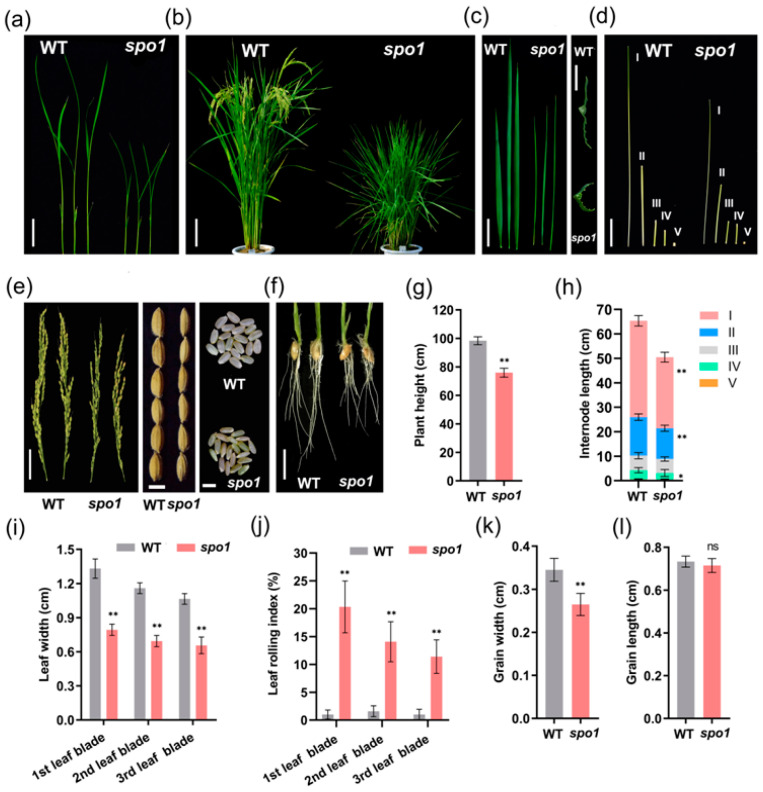
Characterization of WT (Nipponbare) and *spo1* mutant morphology. (**a**) Two-week-old WT and *spo1* seedlings. (**b**) Gross morphology of WT and *spo1* plants at the mature stage. (**c**) Morphology of the top three leaf blades between WT and *spo1* mutant and the transverse sections of WT and *spo1* mature leaves. (**d**) Features of internodes (I, II, III, IV, and V) among WT and *spo1* mutant at the mature stage. (**e**) Panicles, paddy rice grains, and brown rice grains of WT and *spo1* plants. (**f**) Roots of WT and *spo1* plants. (**g**–**l**) Quantification data of plant height (**g**), internode length (**h**), leaf width (**i**), leaf rolling index (LRI) (**j**), grain width (**k**) and length (**l**) of WT and *spo1* mutant at mature stage. At least 12 samples of WT and *spo1* mutant were measured for each. Data are means ± SD, asterisks indicate significant differences according to Student’s *t*-test (* *p* < 0.05; ** *p* < 0.01, ns means no significance). Scale bars: (**a**) 2.5 cm; (**b**) 10 cm; (**c**) 0.35 cm and 6 cm; (**d**) 5 cm; (**e**) 4 cm, 0.35 cm, and 0.4 cm; (**f**) 2 cm.

At the seedling stage, the *spo1* mutant exhibited abnormal phenotypes including reduced plant height and narrowed and curled leaves (Figure 1a). At the mature stage, the *spo1* mutant showed completely different plant architecture, including plant height, leaf length and width, tiller number, and grain size, as compared with the WT (Figure 1b–e). The plant height of *spo1* was 77.18% of that of WT (Figure 1b,g), and the reduced plant height of *spo1* was mainly due to shortened I, II, and IV internodes of the culm (Figure 1d,h). Compared to WT, the *spo1* mutant has more tillers per plant (Figure 1b and Table 1). The width of leaf blades in the *spo1* mutant was significantly reduced (Figure 1c,i) and the length of leaf blades was also significantly reduced in the second and third leaf in *spo1* mutant (Figure 1c and Table 1). Furthermore, the *spo1* mutant exhibited significantly increases in leaf-rolling index (LRI, Figure 1c,j) and leaf angle (Table 1). Associated with the characterization above, it was showed that the *spo1* mutant also has significant reductions including grain width (Figure 1e,k), 1000-grain weight, panicle length, and fertility rate (Table 1) as compared with WT. Moreover, we noticed that the *spo1* mutant exhibited abnormalities in roots (Figure 1f and Table 1) and reduced content of chlorophyll a (Chl a) in leaves (Figure S1). These observations indicated that the *spo1* mutant has small organs and multiple morphological defects.

### 2.2. Map-Based Cloning Revealed That SPO1 Gene Encodes Cellulose Synthase-like D4

**Figure 2:**
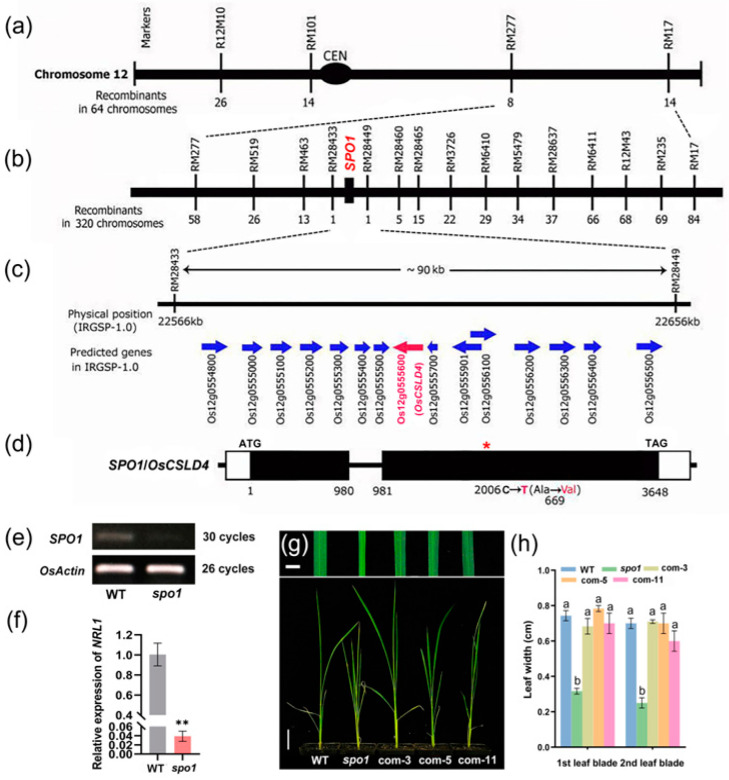
Map-based cloning of *SPO1*. (**a**) Linkage map of the gene *SPO1* on the long arm of chromosome 12. (**b**) Fine-mapping of the *SPO1* locus. The genetic linkage map is derived from 32 F_2_ mutant individuals and 160 F_2_ mutant individuals for fine-mapping. Marker names are above the vertical lines and the number of recombinants is displayed under the vertical lines. (**c**) According to IRGSP1.0 database annotation, the 90-kb region contains 15 annotated genes. (**d**) Gene structure of *SPO1*/*OsCSLD4*/*Os12g0555600* and the corresponding positions of intron and exons. The white boxes indicate the 5′ and 3′ untranslated regions, the black boxes indicate the exons, and the black line between the two black boxes indicate the intron. The start codon (ATG) and the stop codon (TAG) are indicated. The *spo1* mutant has a base substitution (C to T) in the second exon at position 2006 of the coding regions. (**e**) RT-PCR analysis of *SPO1* expression in *spo1*. (**f**) qRT-PCR analysis of *SPO1* expression in *spo1*. Data are means ± SD, asterisks indicate significant differences according to Student’s *t*-test (** *p* < 0.01). (**g**) Genetic complementation of *spo1*. Three representative lines (com-3, com-5, and com-11) of complementation with young plants are shown. (**h**) Statistical analysis of the leaf blades width of WT, *spo1*, and complementary lines (com-3, com-5, and com-11) at seedling stage. Data presented are means ± SD: different letters indicate significant differences between means, according to Duncan’s multiple range test (5% α). Scale bars: (**g**) 0.6 cm and 4 cm, respectively.

To isolate the *SPO1* gene by map-based cloning, we developed a genetic population by crossing the *spo1* mutant with the *indica* cultivar Kasalath. All the F_1_ plants displayed wild-type phenotype. In the F_2_ mapping population, 221 normal individuals and 79 *spo1* individuals were obtained, fitting to the 3:1 Mendel’s separation ratio, indicating that *spo1* was controlled by a single recessive gene. Genetic mapping of the *SPO1* gene using F_2_ mutant individuals revealed that *SPO1* was linked with molecular markers R12M10 and RM101 on rice chromosome 12 (Figure 2a). Using 32 F_2_ mutant individuals, the *SPO1* gene was initially mapped to a region on the long arm of chromosome 12 between molecular markers RM277 and RM17 (Figure 2a). The *SPO1* gene was further fine-mapped to a 90-kb physical region between molecular markers RM28433 and RM28449 by genotyping of 160 F_2_ mutant individuals (Figure 2b). According to gene annotation in the IRGSP 1.0 database (https://rapdb.dna.affrc.go.jp/, accessed on 1 May 2020), there are 15 candidate genes in the 90-kb mapping region (Figure 2c). The candidate *OsCSLD4*/*Os12g0555600*, reported previously, encodes cellulose synthase-like D4 and is essential for normal cell-wall biosynthesis, plant growth, and abiotic resistance [55,56,57,58,59,60,61,62,63]. Gene sequencing analysis revealed that *spo1* contains C to T substitution in the second exon of *OsCSLD4*, resulting in an amino acid substitution from Ala to Val (Figure 2d). RT-PCR and qRT-PCR analyses further showed that the mRNA expression of *SPO1/OsCSLD4* was greatly reduced in the *spo1* mutant (Figure 2e,f). Predictions of protein structure revealed that the *spo1* mutation (C to T substitution) caused a change of OsCSLD4 three-dimensional structure in the direction of rotation (Figure S2a). Then, phylogenetic analysis and multiple sequence alignments were performed among the SPO1/OsCSLD4 and its homologous proteins. The phylogenetic analysis showed that the SPO1/OsCSLD4 protein had the closest relationship with BdCSLD4, ZmCSLD1, and AtCSLD5 (Figure S2b). The result from multiple sequence alignment analysis showed that the amino acid Ala at the *SPO1* mutation site is highly conserved in higher plants (Figure S2c), implying that the amino acid substitution from Ala to Val had a profound effect on the SPO1/OsCSLD4 protein function.

To further confirm *SPO1* function, complementation experiment was performed by genetic transformation of the wild-type *SPO1*/*OsCSLD4* gene into the *spo1* mutant. Compared with that of the *spo1* mutant, the phenotype of complementary transgenic lines (Com-*SPO1*) is similar to that of wild-type plants including increased plant height and reduced leaf rolling (Figure 2g), and increased leaf width (Figure 2g,h). These results indicated that the *spo1* mutant phenotype is caused by the loss-of-function mutation of *SPO1*/*OsCSLD4*. We then overexpressed *SPO1* in Nipponbare. However, there was no significant phenotypic difference between the overexpression lines and wild-type Nipponbare (Figure S3a–i).

### 2.3. The Tissue-Specific and Stressed-Induced Expression Patterns of SPO1

**Figure 3:**
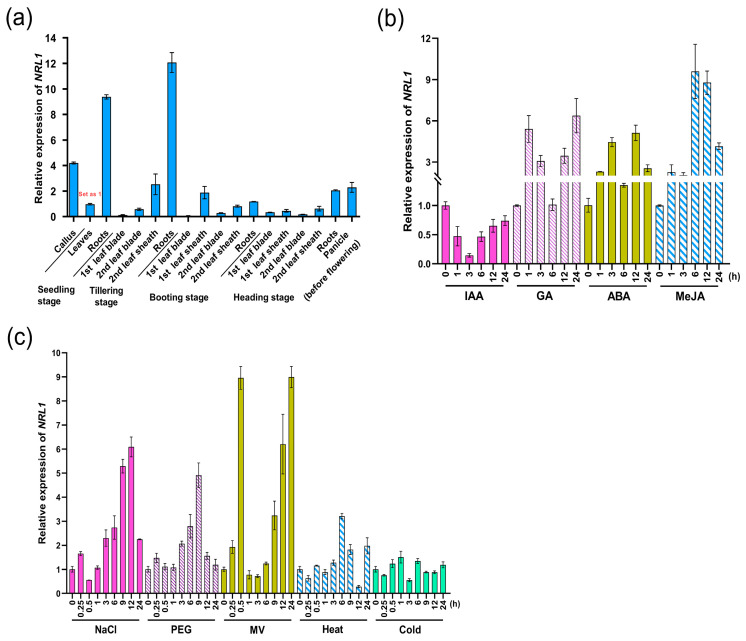
qRT-PCR analysis of the tissue-specific and stressed-induced expression pattern of *SPO1*. (**a**) The tissue-specific expression pattern of *SPO1*. The expression of *SPO1* in leaves at seedling stage was set to 1. (**b**) Expression of *SPO1* in leaves of two-week-old seedlings treated with 100 μM IAA, 50 μM GA, 100 μM ABA, and 100 μM MeJA at different time points. (**c**) Expression of *SPO1* in leaves of two-week-old seedlings treated with 200 mM NaCl, 20%PEG6000, 15 μM MV, heat (42 °C), and cold (4 °C) at different time points. The *OsActin1* gene was used as a control. The data are shown by the mean ± SD with three biological replicates.

We examined the detailed tissue-specific expression patterns by qRT-PCR in different tissues of Nipponbare. It was revealed that the expression of *SPO1* was highly tissue-specific, with the highest expressions in roots at seedling stage and tillering stage of rice plants (Figure 3a). However, the expression level of *SPO1* was relatively low in the leaves at all stages tested (Figure 3a). On the whole, *SPO1* showed higher expression levels in vigorous growth organs.

Analysis of cis-elements in the promoter region of *SPO1* was performed on PlantCARE (http://bioinformatics.psb.ugent.be/webtools/plantcare/html/, accessed on 1 May 2020). This revealed that the *SPO1* promoter contains several elements, such as auxin-responsive element TGA-element, ABA-response element (ABRE), gibberellin-response element TATC-box, and drought-response element MBS, etc., (Table S1), suggesting that *SPO1* might be regulated by hormones and respond to abiotic stress. We then performed the stimulus-induced expression analysis of *SPO1*. On the whole, the *SPO1* showed up- or down-regulation in response to different treatments or stresses. We analyzed the expression of *SPO1* by exogenous applications of hormone IAA, GA, ABA, and MeJA. It was showed that *SPO1* was strongly induced by GA at 1 h but was decreased to a low level at 6 h (Figure 3b). However, the expression of *SPO1* increased again at 12 and 24 h after GA treatment (Figure 3b). Exogenous application of hormone ABA also induced the expression of *SPO1*, but application of hormone IAA significantly decreased the expression of *SPO1* (Figure 3b). The expression of *SPO1* was up-regulated from 1 to 9 h by MeJA treatment (Figure 3b). We further analyzed the expression of *SPO1* under different stress conditions, including NaCl, drought, oxidative, heat, and cold stress. The *SPO1* expressions were progressively induced at 3, 6, 9, and 12 h after treatment with NaCl (Figure 3c). Under drought stress mimicked by PEG-6000 treatment, the expression of *SPO1* was increased at 3, 6, and 9 h (Figure 3c). Under oxidative stress via MV treatment, *SPO1* expression was rapidly increased at 0.5 h, but decreased to the pre-treatment level at 1 h (Figure 3c). The expression of *SPO1* was increased after 6 h by high-temperature stress, but the expression of *SPO1* was not significantly affected under low temperature conditions (Figure 3c).

### 2.4. SPO1 Regulates Leaf Shape, Plant Height, and Grain Size through Affecting Cell Division and/or Cell Expansion

**Figure 4:**
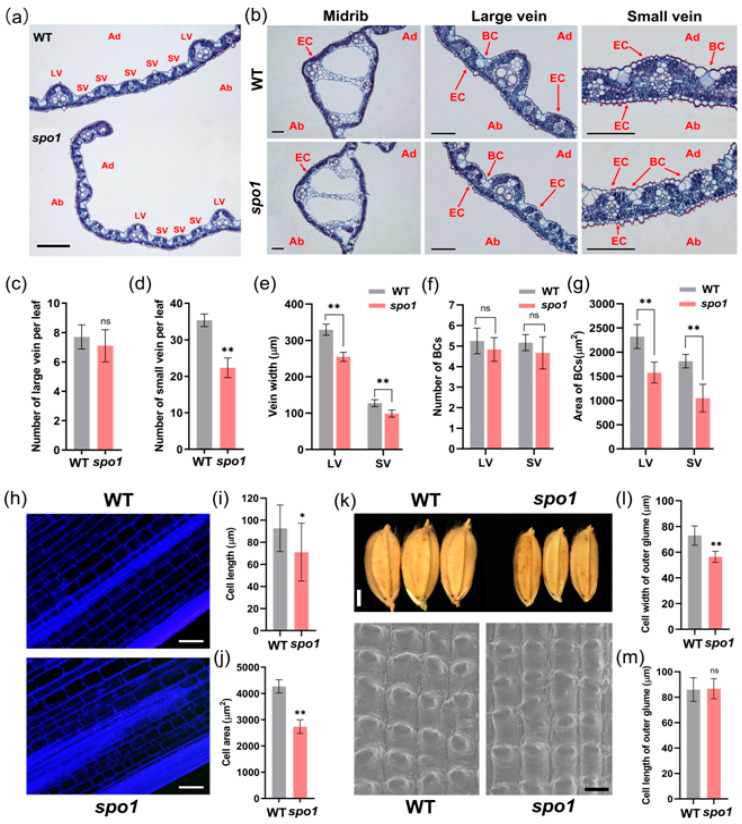
Histological analysis of the leaf blades, stems, and grains between WT and *spo1* mutant plants. (**a**) Cross sections of leaf blades of WT and *spo1* mutant. (**b**) Cross section of midrib, large vein, and small vein at the middle of the second leaf from the top of WT and *spo1* mutant at tillering stage. (**c**–**g**) Statistics analysis of a number of large veins (**c**) and small veins (**d**) per leaf, width of veins (**e**), number of BCs (**f**), area of BCs (**g**). (**h**) Longitudinal sections of stems between WT and *spo1* mutant. (**i**) The length of stem cells. (**j**) The area of stem cells. (**k**) Grain morphology and scanning electron microscopy of the outer surfaces of WT and *spo1* glumes. (**l,m**) Cell width (**l**) and length (**m**) of outer glume of the grain. At least 30 samples of WT and *spo1* mutant were measured for each. Data are means ± SD, asterisks indicate significant differences according to Student’s *t*-test (* *p* < 0.05; ** *p* < 0.01, ns means no significance). Abbreviations: Ab, abaxial surface; Ad, adaxial surface; BC, bulliform cell; EC, epidermal cell; LV, large vein; SV, small vein. Scale bars: (**a**) 500 μm; (**b**) 100 μm; (**h**) 100 μm; (**k**) 0.2 cm and 50 μm.

Because *spo1* showed abnormalities in leaf shape, plant height, and grain size, we therefore conducted cytological characterization on the wild type and the mutant. According to cross-sections on the leaf blade, it was showed that the number of leaf veins, especially small veins, and the mean width of large and small veins were significantly reduced in *spo1* mutant (Figure 4a,c,d,e). The *spo1* mutant also showed a reduced size of bulliform cells (BCs), but no difference in the number of BCs (Figure 4b,f,g). According to the observation on longitudinal sections of culms, it was demonstrated that the cell length and areas were significantly reduced in *spo1* mutant (Figure 4h–j). It is suggested that spikelet hulls can limit grain growth and affect grain size [65]. Because the *spo1* mutant showed a reduction in grain width, we further analyzed the cell width and length of spikelet hulls. According to observation by SEM, it is demonstrated that the width of the longitudinal cells on the outer epidermis of spikelet hulls was significantly reduced in the *spo1* mutant (Figure 4k,l). However, there is no difference in the length of the longitudinal cells on the outer epidermis of spikelet hulls between the WT and *spo1* mutant (Figure 4k,m). Taken together, these data indicated that *SPO1* regulates leaf shape, plant height, and grain size, mainly by affecting cell division and/or cell expansion.

### 2.5. Hormone Contents Were Significantly Altered in the spo1 Mutant

To investigate if hormone-related processes were affected in the *spo1* mutant, we further analyzed hormone contents in the leaf of *spo1* and WT. Various hormones such as IAA, ABA, GA_3_, GA_4_, ZR, and dhZR content are significantly affected. The IAA content is about 2 times lower in *spo1* than in wild-type plants. The ABA content is also decreased in *spo1* compared to WT (Table 2). A 29.84% and 31.6% increase in the content of GA_3_ and GA_4_, respectively, is measured in the *spo1* mutant compared to the wild type (Table 2). Similarly, ZR and dhZR were also increased in the *spo1* mutant (Table 2). These results indicated that the loss-of-function of *SPO1* has a significant impact on hormone contents.

### 2.6. Loss-of-Function of SPO1 Affects the Expression of Leaf-Shape-Related Genes

**Figure 5:**
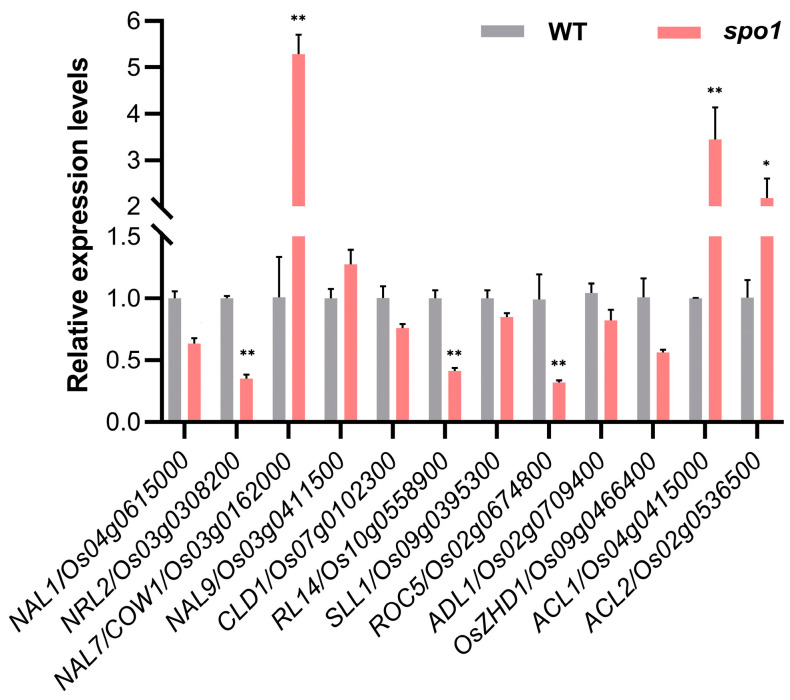
qRT-PCR analysis of the expression of genes related to leaf shape regulation in the *spo1* mutant. Total RNA was extracted from leaves of WT and *spo1* mutant at tillering stage. *OsActin1* gene was used as a control. Data are the mean ± SD with three biological replicates, asterisks indicate significant differences according to Student’s *t*-test (* *p* < 0.05; ** *p* < 0.01).

To further investigate how *SPO1* functions in regulation of leaf development, the expressions of leaf-shape-related genes, such as *NAL1*, *NRL2*, *NAL7*, *NAL9*, etc., were examined by qRT-PCR. The results showed that the loss of *SPO1* gene function resulted in a strong increase in *NAL7*/*COW1* (a flavin-containing monooxygenase gene, related to narrow and curled leaf phenotype) [17], *ACL1*, and *ACL2* (relate to abaxial leaf curling phenotype) [19] expressions in the *spo1* mutant (Figure 5). In contrast, the expressions of *NRL2* (a novel protein with a conserved function, related to regulating leaf width) [12], *ROC5* (homeodomain leucine zipper class IV gene, related to rolled leaf phenotype) [20], and *RL14* (a 2OG-Fe (II) oxygenase family protein gene, related to narrow and rolled leaf phenotype) [22], were significantly down-regulated in the *spo1* mutant (Figure 5). These results showed that the loss-of-function of *SPO1* affects the expression of these genes in regulating leaf morphological development of rice.

### 2.7. Transcriptomic Analysis Revealed SPO1 Plays an Important Role in Hormone, Cell Cycle, and Cell Wall Formation Pathways

**Figure 6:**
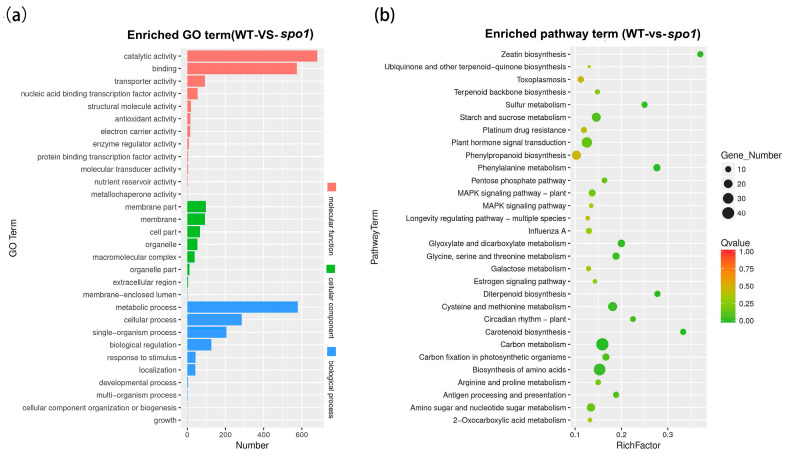
Transcriptomic analysis of *spo1* mutant. (**a**) Gene Ontology (GO) enrichment analysis of DEGs in *spo1*. (**b**) KEGG pathway enrichment of DEGs in *spo1*. Leaves of *spo1* mutant and wild-type plants in the tillering stage are used as test samples.

To further understand the molecular mechanism of *SPO1* in the regulation of organ size, especially leaf shape, RNA-seq analysis was performed with the leaf blade of the *spo1* mutant and wild-type plants at the tillering stage. A total of 2379 differentially expressed genes (DEGs) including 848 down-regulated genes and 1531 up-regulated genes were identified by RNA-seq (Figure S4, Table S3). Gene ontology (GO) enrichment analysis revealed that the 2379 DEGs were significantly enriched in 10 terms at the biological process category, 8 terms at the cellular component category, and 12 terms at the molecular function category (Figure 6a). In the biological process category, the most enriched GO term is metabolic process (Figure 6a). In the cellular component category, the top two most enriched GO terms are the membrane part and membrane (Figure 6a). In the molecular function category, the top three most enriched GO terms include catalytic activity, binding, and transporter activity (Figure 6a). According to the Kyoto Encyclopedia of Genes and Genomes (KEGG) pathway enrichment analysis on the DEGs, the top five most enriched pathways are biosynthesis of amino acids, carbon metabolism, plant hormone signal transduction, starch and sucrose metabolism, and cysteine and methionine metabolism (Figure 6b).

According to KEGG enrichment analysis, plant hormone signal transduction is one of the most enriched pathways. We found a great number of genes related to auxins, GAs, and CKs pathways were affected in the *spo1* mutant (Table 3). It is demonstrated that auxin response factor (*OsARF11*/*LOC_Os04g56850*), auxin-responsive SAUR gene (*LOC_Os02g07110*), auxin receptor (*OsAFB6*/*LOC_Os03g08850*), auxin efflux transport carrier (*OsPIN5a*/*LOC_Os01g69070*), and indole-3-acetic acid-amido synthetase (*OsMGH3*/*LOC_Os07g40290*) were significantly up-regulated in the *spo1* mutant, but the *AUX1*/*LAX* (*OsAUX4*/*LOC_Os10g05690*, *OsAUX3*/*LOC_Os05g37470*) and two auxin-responsive Aux/IAA genes (*LOC_Os02g56120*/*LOC_Os01g53880*) and IAA synthesis gene *OsYUCCA6* (*LOC_Os07g25540*) were significantly down-regulated in the *spo1* mutant (Table 3). Genes involved in GA-related pathways, such as three genes encoding gibberellin oxidase (*OsGA2ox5*/*LOC_Os07g01340*, *OsGA20ox2*/*LOC_Os01g08220*, *CYP714B2*/*LOC_Os03g21400*), one gene encoding gibberellin 3β-hydroxylase (*OsGA3ox2*/*LOC_Os01g08220*), and two genes encoding gibberellin receptors *GID1L2* (*LOC_Os07g44900*, *LOC_Os09g28230*) were significantly up-regulated in the *spo1* mutant (Table 3). Two genes encoding UDP-glucosyltransferase (*LOC_Os05g08480*, *LOC_Os07g30610*), two genes belonging to the cytokinin oxidase/dehydrogenase family (*OsCKX3*/*LOC_Os10g34230*, *OsCKX4*/*LOC_Os01g71310*), type-A response regulator gene (*OsRR6*/*LOC_Os04g57720*), and cytokinin receptor (*OHK4*/*LOC_Os03g50860*) were significantly up-regulated in the *spo1* mutant compared to the wild type (Table 3). These results indicated that the loss of *SPO1* gene function affected auxins, GAs, and CKs pathways.

In addition, many of the genes associated with cell cycle and cell wall formation were significantly differently expressed in the *spo1* mutant. For example, two genes (*LOC_Os07g30240*, *LOC_Os12g04980*) involved in meiosis were up-regulated in *spo1* and one gene (*LOC_Os05g41880*) related to meiotic reciprocal recombination was down-regulated in the *spo1* mutant (Table 3). Four genes (*LOC_Os04g53680*, *LOC_Os01g13260*, *LOC_Os05g33040*, *LOC_Os09g38768*) encoding cell cycle proteins were differentially expressed in the *spo1* mutant (Table 3). Moreover, a large number of genes related to cell-wall-related pathways were affected in the *spo1* mutant (Table 3). For example, six genes involved in cell wall formation had significantly altered expressions in the *spo1* mutant. A NAC transcription factor OsSWN3 (*LOC_Os08g01330*) and *OsCAD3* (*LOC_Os10g29470*), which participate in cell wall lignin biosynthesis, were significantly up-regulated in the *spo1* mutant. A R2R3-type MYB transcription factor (*LOC_Os04g50770*) and a NAC transcription factor OsSWN2 (*LOC_Os08g02300*), both of which regulate the cell wall cellulose biosynthesis pathway, were down-regulated in the *spo1* mutant. The NAC transcription factor *OsSWN1* (*LOC_Os06g04090*) and the 2OG-Fe (II) oxygenase family protein *RL14* (*LOC_Os10g40960*), which regulate secondary wall biosynthesis, were down-regulated in the *spo1* mutant. These results indicated the loss of *SPO1* gene function affected the processes associated with cell cycle and cell wall formation pathways.

## 3. Discussion

In this study, we characterized a *spo1* mutant that exhibited small plant and organ size, including reduced plant height and root length, narrow and rolled leaf, and slender grain, etc. Through map-based cloning and genetic transformation, *spo1* was identified as a novel allele of *OsCSLD4*, which belongs to the cellulose synthase-like superfamily and functions in cell wall formation, plant growth, and abiotic resistance based on previous functional studies [55,56,57,58,59,60,61,62,63]. Our investigation indicated that *SPO1* may control plant and organ size by modulating cell division and expansion possibly mediated by the effects of multiple hormone pathways on cell wall formation.

The *spo1* mutant formed narrow and rolled leaves (Figure 1a–c) and showed vascular bundles and bulliform cells (BCs) defects in leaf blade (Figure 4a–g), indicating the *SPO1* may act as an important factor influencing rice leaf morphology. Previous studies have shown that changes in leaf vein patterns can lead to leaf narrowing, for example, *NAL1* [8], *NAL2* [9,66], *NAL3* [9,66], *NAL9* [10], and *NAL11* [11] affect leaf width by controlling the number of veins. Mutation of *NRL22* reduces vein width in both large and small veins compared to the wild type, thereby producing narrow leaf phenotypes [16]. In this study, the *spo1* leaf showed a reduced number and width of veins, suggesting that *SPO1* is involved in the formation of vascular bundles in the leaf. Furthermore, among the genes tested to regulate leaf morphology, *NAL7* is strongly up-regulated in the *spo1* mutant (Figure 5). Loss of function of *NAL7*, which encodes a member of the rice YUCCA gene family that regulates auxin biosynthesis, results in narrow and curled leaves and altered IAA content [17]. In addition, the expression levels of *RL14*, *ROC5*, *ACL1*, and *ACL2* appear to be down- or up-regulated in the *spo1* mutant (Figure 5). These four genes cause leaf curling by directly or indirectly regulating the size or number of BCs. The *NRL2*, which interacts with *RL14* and has a higher cellulose content and lower lignin content than the WT, is also down-regulated in the *spo1* mutant. These results suggest that there are certain functional connections between *SPO1* and these tested genes in regulating leaf morphology. Therefore, the function of the *SPO1* gene is required for the normal formation of veins and BCs in the leaf blade.

Plant hormones such as auxin, gibberellin (GA), cytokinin (CK), and abscisic acid (ABA) have various functions in plant growth and development [67]. Changes in hormone levels or homeostasis in plant may affect plant architecture and organ size. Several genes that regulate plant architecture and organ size in relation to hormone pathways have been identified and characterized in rice. For instance, the narrow leaf mutant *nal7* [17], *osarf24* [68], and tryptophan-deficient dwarf mutant *tdd1* [39], which are all related to the auxin pathway. The dwarf mutants *gid1* [69], *gid2* [70], *slr1* [71], *d18* [72], and *sd1* [73], and the small seeds mutants *oscbl5* [32] and *sgsd3* [74], are related to the gibberellin pathway. In the present study, many traits related to organ size, including narrow leaf, dwarf plant, and small grain characteristics, may be associated with auxin and GAs. The content of IAA is decreased, but the contents of GA_3_ and GA_4_ are increased in the *spo1* mutant as compared to the wild type (Table 2). Rice YUCCA (YUC) flavin-containing monooxygenase-encoding gene *OsYUC11* has been shown to be a key factor for auxin biosynthesis. The *osyuc11* showed reduced seed size and increased chalkiness, accompanied by reduced IAA level [75]. The *gid1* is a GA-insensitive mutant, which had a severe dwarf phenotype with wide, dark-green leaf blades and increased GA levels compared with the wild type [69]. These results are consistent with the phenotype in the *spo1* mutant. Given this knowledge, the *spo1* mutant may be an IAA-deficient and GA-insensitive mutant. Our RNA-seq data revealed that the expression of auxin-responsive genes (*OsIAA6*, *OsIAA9*), IAA synthetic pathway gene (*OsYUCCA6*), and auxin influx transporter genes (*OsAUX3*, *OsAUX4*) were lower in the *spo1* mutant compared to the wild type. Efflux carriers of polar auxin transporter (*OsPIN5a*), auxin-signaling F-Box gene (*OsAFB6*), and indole-3-acetate beta-glucosyl transferase were up-regulated in the *spo1* mutant compared to the wild type. However, auxin response factor, *OsARF11*, auxin-responsive SAUR gene, *OsSAUR6* and tryptophan amino transferase, and *FIB* were up-regulated in the *spo1* mutant compared to the wild type. In addition, the expressions of genes related to the GA pathway (*OsGA2ox5*, *D18*, *SD1*, *CYP714B2,* and two GA receptor-like genes (*GID1L2*)) are higher in the *spo1* mutant compared to the wild type. *D18* and *SD1* participate in the synthesis of GA and exhibit plant dwarfism [72,73]. Overexpression of *CYP714B2* can lead to semi-dwarfism in Arabidopsis plants and increase the content of 13-OH type GAs [76]. Overexpressing *OsGA2ox5*, a gene involving in the GA catabolic pathway, exhibited dominant dwarf phenotype in rice [41]. Therefore, the function of *SPO1* may be related to the aforementioned genes and loss of *SPO1* function may disrupt auxin and GA homeostasis and signaling, thus resulting in smaller organ size in the *spo1* mutant. The increasing transcription levels of *OsARF11*, *OsSAUR6*, *FIB*, and *OsGA2ox5* in the *spo1* mutant may indicate that their regulations are required for plants to maintain the endogenous IAA and GAs levels stably. Moreover, the organ size is sustained by the coordination of the two basic cellular processes of cell division and cell expansion, which determine cell numbers and cell sizes, respectively [4,5]. The leaf morphology is also influenced by cell proliferation and cell expansion [14,77]. In fact, previous studies have revealed that *sle1*, the allelic mutant of *spo1*, had reduced cell proliferation beginning at the P3 primordial stage, resulting in narrow leaf blades [59]. Consistent with this finding, according to our RNA-seq data, the transcripts levels of meiotic recombination genes (*OsMSH4*, *OsDMC1A*, *OsMSH5*) and cyclin genes (*CycP1;1*, *CycA1;1*, *OsCYCP1;1*) that control cell cycle processes were changed to varying degrees in the *spo1* mutant. The results suggest that *SPO1* affects the cell proliferation process by altering the expression of these cell cycle genes, and ultimately affecting rice architecture and organ size including leaf development. In addition to changes in leaf morphology, the *spo1* mutant also forms a dwarf and slender grain phenotype, which contributes to small organs (Figure 1a,b,d,e). The longitudinal sections of the shortened internodes in the *spo1* mutant exhibited a reduction in the cell length and area (Figure 2h–j). In addition, the width of the longitudinal cells on the outer epidermis of the spikelet hulls was significantly reduced in the *spo1* mutant, as determined by SEM observation (Figure 2k–m). These findings indicated that *SPO1* regulates plant height and grain size by the progress of cell expansion. Auxin and GAs are mainly responsible for cell division and expansion [36,37]. *NAL1* regulates cell division and affects polar auxin transport and vascular patterns, and mutation of *NAL1* resulting in a reduction in leaf blade width [8,26]. Activation of rice *BIG GRAIN1* (*BG1*), which is involved in auxin transport, results in large grains due to increased cell proliferation and cell expansion in spikelet hulls [44]. BC12/GDD1, a kinesin-like protein with transcription regulation activity, mediates cell elongation by regulating the GA biosynthesis pathway in rice [78]. Consistent with these, it is very likely that altered expressions of genes relating to auxins and GAs pathway in the *spo1* mutant were owing to disrupted auxin and GA homeostasis and signaling, which are involved in the cellular processes of cell division and cell expansion. In addition to auxin and GAs, CKs also mediate organs size in plant growth and development including cell division and cell expansion [79]. In the *spo1* mutant, the expression of *OsCKX4* and *OsCKX3* was increased. Cytokinin oxidase/dehydrogenases (CKXs) are a group of enzymes that regulate oxidative cleavage to maintain cytokinin homeostasis [80]. *OsRR6* acts as a negative regulator of CK signaling, the overexpression of which leads to dwarf phenotypes [81]. Therefore, it was possible that abnormal CK signaling in the *spo1* mutant might also be attributed to altered morphological characters, such as plant height and grain size.

In fact, the role of hormones in plant growth cannot be easily summarized by a linear signaling pathway. Some hormones such as ABA, and BR are also involved in regulating organ size and plant architecture through crosstalk and integration with multiple hormone pathways [49,82]. For instance, *OsIAA1* may play important roles in the crosstalk between auxin and the BR signaling pathways during plant morphogenesis. *OsIAA1* overexpression in transgenic plants showed decreased plant height and loose plant architecture due to decreased auxin sensitivity and increased BR sensitivity [50]. In the present study, *SPO1* may be induced to varying degrees by different hormones, and hormone levels such as IAA, GAs, CKs, and BR were also changed in the *spo1* mutant. In addition, genes involved in different hormone pathways have been identified by RNA-seq. According to these results, we suggest that *SPO1* may regulate hormone homeostasis by integrating multiple hormone pathways, thereby regulating cell division and expansion, and ultimately regulating plant architecture and organ size.

Previous studies have reported that *OsCSLD4* plays an important role in cell wall formation by altering xylan and cellulose content, and indeed the cellulose content was reduced in *OsCSLD4* mutant [55]. In this study, a large number of DEGs involved in cell wall formation were identified in *spo1* mutant (Table S3). For instance, OsSWN3 (*LOC_Os08g01330*) and *OsCAD3* (*LOC_Os10g29470*) which participate in cell wall lignin biosynthesis, a R2R3-type MYB transcription factor (*LOC_Os04g50770*) and a NAC transcription factor OsSWN2 (*LOC_Os08g02300*) which regulate the cell wall cellulose biosynthesis pathway, and a NAC transcription factor *OsSWN1* (*LOC_Os06g04090*) and 2OG-Fe (II) oxygenase family member *RL14* (*LOC_Os10g40960*) which regulate secondary wall biosynthesis, were differentially expressed in *spo1* mutant. This finding suggests that *SPO1* is involved in regulating the synthesis of cell walls. Studies have shown that a decrease in cellulose content can lead to weakened mechanical support and increased leaf angle [83]. In breeding, rational dense planting is advocated to increase yield, while increased leaf angle requires more space for growth, which affects yield and leads to decreased fertility rate and biomass. The *spo1* mutant has significantly reduced leaf width, which may affect its photosynthetic capacity and overall growth. This may also be the reason for the decreased fertility rate. Root length was significantly shorter in the mutant, which may lead to inadequate nutrient uptake and thus affect yield. *SPO1* has a very obvious “one-cause-multiple-effect” role, suggesting that this gene plays an important role at all stages of rice development. The data reported here that *SPO1* is allelic with *SPO1*/*OsCSLD4*. As shown in Figure S5, the *spo1* mutant has one single nucleotide substitution (C2006T) that is same as *dnl1* and *nd1*, but each of their mutation positions is different. Additionally, the mutated amino acids are different between the *spo1* and *dnl1* mutant. Therefore, we demonstrated that *spo1* is a novel allele of *SPO1*/*OsCSLD4*. The mutated amino acid Ala in *spo1* is highly conserved in CSLD members of plants (Figure S2c); the amino acid Ala was mutated to Val by the single base mutation in the *SPO1*/*OsCSLD4* gene, but the polarity of the amino acids did not change. However, it led to severe mutations in multiple phenotypes, indicating the importance of this site. In addition, *SPO1*/*OsCSLD4* has noteworthy biological functions in the regulation of plant architecture and organ size in rice.

## 4. Materials and Methods

### 4.1. Plant Materials and Growth Conditions

The *spo1* mutant was isolated from the rice cultivar Nipponbare (*Oryza sativa* L. *japonica*) by mutagenesis with ethyl methanesulfonate. Overexpressing and complementary transgenic plants were obtained by infecting the rice calli of the Nipponbare variety and the *spo1* mutant, respectively, with *Agrobacterium tumefaciens* carrying related vectors. All plant materials were grown in pots or in paddy fields under natural conditions and in a greenhouse with a 14 h light (30 °C)/10 h dark (26 °C) cycle, and with 70–80% relative humidity.

### 4.2. The Mapping of the SPO1 Gene

For genetic mapping, the *spo1* mutant was crossed with the rice cultivar Kasalath (*Oryza sativa* L. *indica*). We identified 318 mutant individuals from the F_2_ population. Preliminary genetic mapping was performed by genotyping of 32 F_2_ mutant plants with SSR markers (Table S2). For the fine-mapping of *spo1*, 20 new SSR markers was designed and 160 mutant individuals of the F_2_ population was used. To identify the mutation site, a candidate gene was amplified using genomic DNA extracted from the *spo1* mutant and Nipponbare and sequenced. The primer sequences used in the fine-mapping and candidate gene analyses are listed in Table S2.

### 4.3. Vector Construction and Plant Transformation

For the functional complementation test, the full-length CDS of *SPO1* was cloned into the binary vector *pUN-1301*. The recombinant plasmids were introduced into the *spo1* mutant using the Agrobacterium tumefaciens-mediated transformation method, as described previously [25]. To generate the overexpression transgenic lines of *SPO1*, the full-length CDS of *SPO1* was cloned into the binary vector *p35S-1301-GFP*. The recombinant plasmids were introduced into wild-type Nipponbare calli by the Agrobacterium-mediated transformation method [25]. The positive transgenic plants above were identified by the evaluation of hygromycin resistance and GUS array. The primer sequences used are listed in Table S2.

### 4.4. Microscopy Analysis

The fresh culm segments and the second top leaves of WT and *spo1* at tilling stage were collected and immediately fixed in formaldehyde alcohol acetic acid (FAA) solution at 4 °C overnight. Microscopic analysis was performed using both hand-cut sections and paraffin sectioning methods [12,29]. The sections were examined and photographed using an Olympus BX51 microscope (Tokyo, Japan). At least three independent experiments were carried out for each analysis. The number and area of the bulliform cells were measured by using the ImageJ software (https://imagej.net/downloads, accessed on 1 May 2020).

### 4.5. RNA Extraction and Gene Expression Analysis

To study the tissue expression patterns of *SPO1*, total RNA was extracted from various tissues of cultivar Nipponbare at different developmental stages using RNAiso^TM^ Plus Trizol (TaKaRa, Dalian, China). For expression analyses of *SPO1* gene under various hormone treatment and stress conditions, total RNA was extracted from 2-week-old Nipponbare seedlings by using the above method. For the analysis of transcripts of *SPO1* and some leaf-shape-related genes, total RNA was isolated from leaf blades of WT, *spo1* mutant, and transgenic plants in the tillering stage using the same method. First-strand cDNAs were synthesized from 2.0 μg total RNA using Integrated First-strand cDNA Synthesis Kit (One-Step gDNA Remover) (DiNing, Beijing, China) following the manufacturer’s instructions. RT-PCR analysis was conducted with the gene-specific primers. The rice *OsActin1* gene was used as an internal control in RT-PCR analysis. The qRT-PCR was performed on a Bio-Rad CFX96 real-time system (Bio-Rad Laboratories Inc., Hercules, CA, USA) with ChamQ Universal SYBR qPCR Master Mix (Vazyme, Beijing, China) following the manufacturer’s instructions. The relative expression level of the target gene was obtained by normalization to internal reference gene *OsActin1*. Each set of experiments had three biological replicates, and the quantitative variation was evaluated by the 2^−ΔΔCt^ relative quantification method [84]. The primer sequences were listed in Table S2.

### 4.6. Measurement of Endogenous Hormone Content

Endogenous hormone content of samples was measured according to the enzyme-linked immunosorbent assay (ELISA) method described by Li et al. (2021) [85] with some modifications. Approximately 500 mg leaves of WT and *spo1* plants at tilling stage were ground to a fine powder with liquid nitrogen for testing. At least three independent replicates were tested.

### 4.7. RNA-seq Analysis

The leaves of WT and *spo1* plants at tillering stage were collected, with three biological replicates of each to test. The experimental procedures for RNA-seq and data analysis were performed according to the description given by Li et al. (2021) [85].

### 4.8. Measurements

For testing leaf rolling index (LRI), Lw (expand the leaf blade and determine the greatest width) and Ln (the natural distance of leaf blade margins at the same position) were measured. The LRI was calculated as LRI (%) = (Lw − Ln)/Lw × 100 [29]. The chlorophyll content was measured as described by Chen et al. (2013) [86], and the method slightly modified. An equal weight of the second top leaf tissues was ground in 95% ethanol under dark conditions and filtered. Then, the concentrations of chlorophyll were measured at 665 nm and 649 nm. The contents of chlorophyll a and chlorophyll b were calculated according to the following formula: Ca = 13.95A_665_ − 6.88A_649_, Cb = 24.96A_649_ − 7.32A_665_.

### 4.9. Phylogenetic Analysis and Protein Structure Prediction

The homologous proteins of *SPO1* were identified by the Blastp search program of the National Center for Biotechnology Information (NCBI https://www.ncbi.nlm.nih.gov/ and Phytozome 13 https://phytozome-next.jgi.doe.gov/, accessed on 1 May 2020). All of the sequences in the CSLD subfamily from *Oryza sativa*, *Arabidopsis thaliana*, *Brachypodium distachyon*, and *Zea mays* were used to construct a phylogenetic tree using MEGA 7.0 software by the neighbor-joining (NJ) method with 1000 bootstrap replications. Multiple sequence alignments were conducted using DNAMAN version 6.0 software. The three-dimensional structure of proteins was constructed with Phyre2 software (http://www.sbg.bio.ic.ac.uk/~phyre2/html/, accessed on 1 October 2023) using default parameters.

## Figures and Tables

**Table 1:** Comparison differences of some agronomic traits between WT and *spo1* mutant.

Traits	WT	*spo1*
Flag leaf length (cm)	33.38 ± 3.46	30.63 ± 4.44
Second leaf length (cm)	42.67 ± 1.94	37.81 ± 1.25 **
Third leaf length (cm)	42.13 ± 1.64	33.44 ± 2.44 **
Tillering number	12 ± 2.23	25 ± 3.91 **
Leaf angle of flag leaf blade (°)	9.38 ± 1.77	18.5 ± 7.63 *
Leaf angle of second leaf blade (°)	9.25 ± 1.39	18.75 ± 7.03 **
Leaf angle of third leaf blade (°)	12.38 ± 2.33	25.13 ± 9.26 **
Fertility rate (%)	82.55 ± 6.92	55.29 ± 7.09 **
1000-grain weight (g)	25.08 ± 0.24	16.53 ± 0.22 **
Panicle length (cm)	23.26 ± 0.73	21.14 ± 1.15 **
Number of adventive roots	14 ± 2.15	12 ± 2.28 *
Primary root length (cm)	8.72 ± 0.83	5.87 ± 0.86 **

The values were shown as mean ± SD (n ≥ 20). Single asterisk (*) indicates that the difference between the WT and *spo1* is statistically significant at *p* < 0.05. Double asterisk (**) indicates that the difference between the WT and *spo1* is statistically significant at *p* < 0.01.

**Table 2:** Hormone content of the wild type (WT) and *spo1* mutant.

Plant Hormone	WT (ng/g FW)	*spo1* (ng/g FW)
IAA	67.854 ± 5.086	37.220 ± 1.185 **
ABA	114.697 ± 7.687	90.670 ± 6.971 **
GA_3_	0.861 ± 0.136	1.118 ± 0.054 **
GA_4_	0.905 ± 0.051	1.316 ± 0.097 **
ZR	2.652 ± 0.075	3.042 ± 0.132 **
dhZR	0.814 ± 0.035	1.002 ± 0.035 **

The values were shown as mean ± SD (n ≥ 3). Double asterisk (**) and indicates that the difference between the WT and *spo1* is statistically significant at *p* < 0.01.

**Table 3:** Differentially expressed genes associated with multiple hormone pathways, cell cycle, and cell wall formation.

Gene ID	Gene Description	log2 Fold Change	*p*-Value	Regulation
Genes involved in auxin pathway				
*LOC_Os04g56850*	Auxin response factor, *OsARF11*	2.56219	2.90 × 10^−6^	Up
*LOC_Os01g59110*	Similar to indole-3-acetate beta-glucosyl transferase	2.0965	1.48 × 10^−18^	Up
*LOC_Os01g69070*	Similar to Efflux carrier of polar auxin transport, *OsPIN5a*	1.69253	7.39 × 10^−10^	Up
*LOC_Os03g08850*	Auxin receptor; auxin-signaling F-Box (AFB) gene, *OsAFB6*	1.42947	6.45 × 10^−10^	Up
*LOC_Os02g07110*	OsSAUR6-Auxin-responsive SAUR gene family member	1.39592	1.42 × 10^−5^	Up
*LOC_Os01g07500*	Tryptophan amino transferase, *FIB*; *OsTAR2*; *OsTAA1*	1.34337	2.07 × 10^−7^	Up
*LOC_Os10g05690*	AUX1/LAX gene; auxin carrier; auxin influx transporter, *OsAUX4*	−2.65314	1.54 × 10^−6^	Down
*LOC_Os05g37470*	AUX1/LAX gene; auxin carrier; auxin influx transporter *OsAUX3*; *qGL5*	−1.42293	0.00015	Down
*LOC_Os02g56120*	OsIAA9-Auxin-responsive Aux/IAA gene family member	−1.57906	0.00380	Down
*LOC_Os01g53880*	Auxin-responsive Aux/IAA gene family member, *OsIAA6*	−1.4906	1.74 × 10^−6^	Down
*LOC_Os07g25540*	IAA synthetic pathway gene, *OsYUCCA6*	−1.32218	0.00781	Down
Genes involved in GA pathway				
*LOC_Os07g01340*	Gibberellin 2-oxidase gene, *OsGA2ox5*	2.34471	4.40 × 10^−11^	Up
*LOC_Os01g08220*	Gibberellin 3β-hydroxylase, GA metabolism, *d18*; *OsGA3ox2*	1.38532	4.77 × 10^−5^	Up
*LOC_Os01g66100*	*Semidwarf-1*; *gibberellin 20-oxidase gene*, *sd1*, *OsGA20ox2*	1.30841	0.00011	Up
*LOC_Os03g21400*	Cytochrome P450 gene; gibberellin 13-oxidase, GA homeostasis, *CYP714B2*	1.27219	1.42 × 10^−11^	Up
*LOC_Os07g44900*	Gibberellin receptor *GID1L2*	1.48675	1.57 × 10^−10^	Up
*LOC_Os09g28230*	Gibberellin receptor *GID1L2*	1.28099	1.08 × 10^−12^	Up
Genes involved in CK pathway				
*LOC_Os05g08480*	UDP-glucuronosyl/UDP-glucosyltransferase family protein, cytokinin-O-glucosyltransferase 1	3.16484	2.15 × 10^−22^	Up
*LOC_Os07g30610*	UDP-glucuronosyl/UDP-glucosyltransferase family protein, cytokinin-O-glucosyltransferase 2	1.29078	3.37 × 10^−7^	Up
*LOC_Os01g71310*	Cytokinin oxidase/dehydrogenase family gene, *OsCKX4*; *REN1*	2.1701	6.74 × 10^−12^	Up
*LOC_Os10g34230*	Cytokinin oxidase/dehydrogenase, *OsCKX3*	1.19109	0.00044	Up
*LOC_Os04g57720*	A-type response regulator gene, *OsRR6*	1.19145	6.26 × 10^−5^	Up
*LOC_Os03g50860*	Cytokinin receptor family, *OHK4*	1.12033	9.44 × 10^−8^	Up
Genes related to cell cycle processes				
*LOC_Os07g30240*	Meiotic recombination; MutS-homolog family gene; ZMM protein, *OsMSH4*	2.89183	2.12 × 10^−12^	Up
*LOC_Os12g04980*	Homologous pairing aberration in rice meiosis; meiosis-specific DNA recombinase, *OsDMC1A*; *DMC1A*	1.72769	0.00218	Up
*LOC_Os04g53680*	Rice cyclin gene, *CycP1;1*	1.44644	9.56 × 10^−9^	Up
*LOC_Os01g13260*	Rice cyclin gene, *CycA1;1*	−1.24606	0.00589	Down
*LOC_Os05g33040*	P-type cyclin gene, *OsCYCP1;1*; *CycP3;1*	−1.27283	4.56 × 10^−5^	Down
*LOC_Os05g41880*	Meiotic reciprocal recombination; MutS-homolog gene; ZMM protein, *OsMSH5*	−1.23514	0.00798	Down
*LOC_Os09g38768*	Cell cycle control protein	−1.16037	0.00016	Down
Genes related to cell wall formation				
*LOC_Os08g01330*	NAC Transcription Factor, affects the synthesis of cellulose in the secondary wall, *OsSWN3*; *NAC31*	2.32202	1.28 × 10^−6^	Up
*LOC_Os10g29470*	*Cinnamyl alcohol dehydrogenase 3*, participates in lignin biosynthesis, *OsCAD3*	1.16085	2.48 × 10^−9^	Up
*LOC_Os04g50770*	R2R3-type MYB family transcription factor, regulation of cellulose biosynthesis during secondary cell wall formation	−2.49826	1.01 × 10^−7^	Down
*LOC_Os06g04090*	NAC transcription factor, regulation of secondary wall biosynthesis by affects the content of lignin, *OsSWN1*	−1.25174	0.00072	Down
*LOC_Os10g40960*	2OG-Fe (II) oxygenase family protein, regulating the formation of secondary cell walls by affecting their components, *RL14*	−1.09748	3.86 × 10^−6^	Down
*LOC_Os08g02300*	NAC Transcription Factor, *OsSWN2*; *NAC29*, affects cellulose synthesis	−1.05195	0.00827	Down

## Data Availability

The data presented in this study are available in the article and Supplementary Materials.

## Supplementary Materials

The following supporting information can be downloaded at: https://www.mdpi.com/article/10.3390/ijms242316974/s1.
