# Supplementary material for: SMALL PLANT AND ORGAN 1 (SPO1) Encoding a Cellulose Synthase-like Protein D4 (OsCSLD4) Is an Important Regulator for Plant Architecture and Organ Size in Rice
Source: Int J Mol Sci. 2023 Nov 30;24(23):16974. doi: 10.3390/ijms242316974 (PMC10707047; doi:10.3390/ijms242316974)
Supplement: Supplementary file 1 [file ijms-24-16974-s001.zip › Figures S1-5 and Tables S1 and S2--ijms-2714925.pdf]

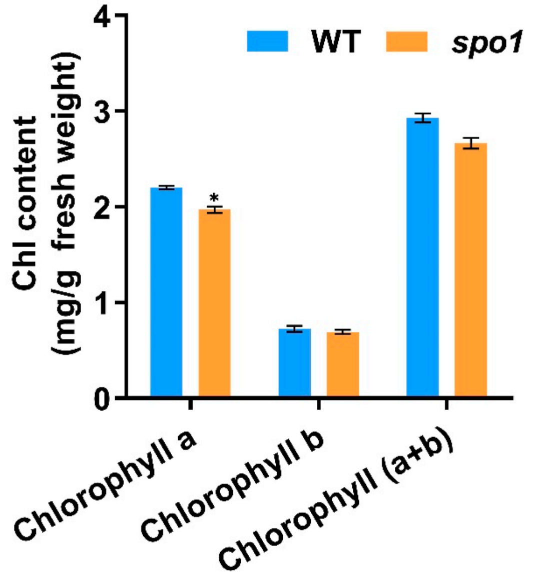

Figure S1. Characterization of pigment content of *spo1* mutant. The pigment contents of second leaf from the top at tillering stage. Pigment contents data are mean ± SD (n = 4), asterisks indicate significant differences according to Student's t-test (*P < 0.05).

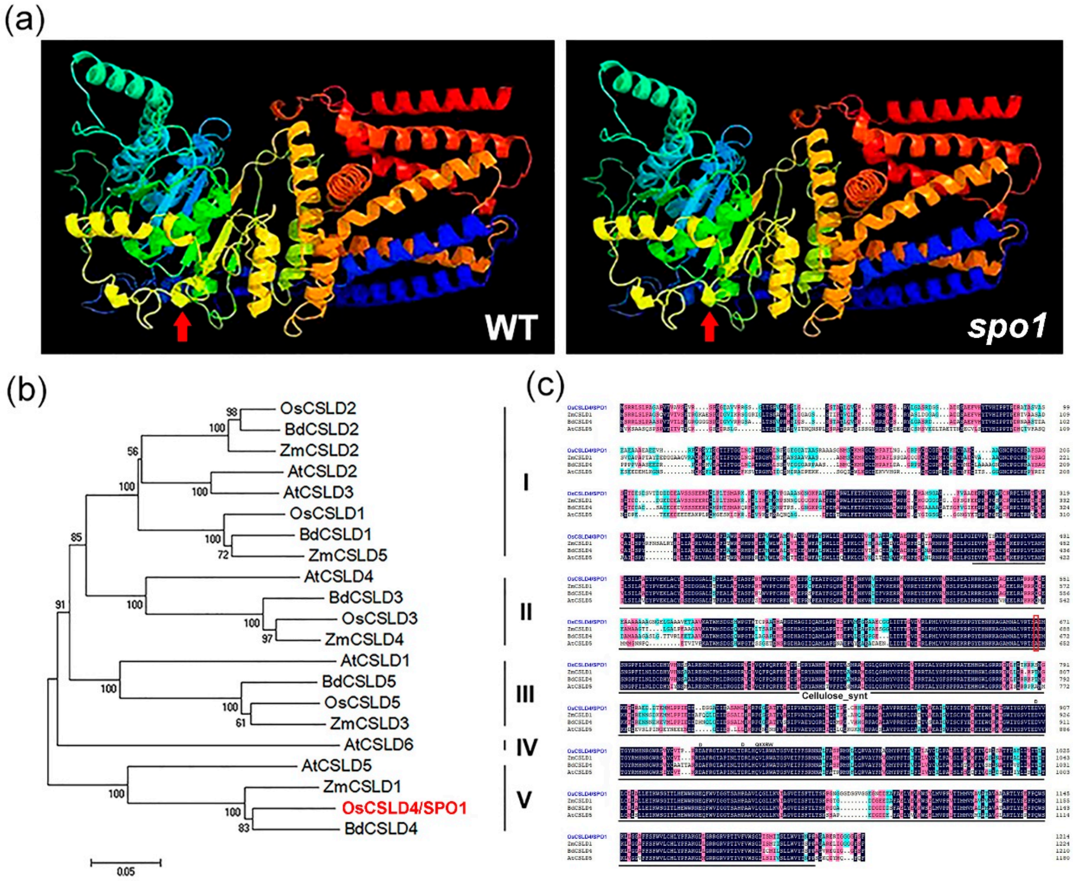

Figure S2. *SPO1* encodes a cellulose synthase-like protein D4. (a)The three-level structure prediction model of WT and *spo1* protein. The arrow is the position of the structural change. (b) Phylogenic tree of SPO1 and its highly similar proteins of CSLD family members among other species. Numbers at each node represent bootstrap support. The scale bar indicates the genetic distance based on branch length.

(c)Multiple sequences alignment of Oryza sativa Japonica Group SPO1/OsCSLD4 (Os12g0555600), BdCSLD4 (XP_003575482.1), AtCSLD5 (NP_171773.1), ZmCSLD1 (GRMZM2G015886). The amino acids showing identity are shaded dark blue, whereas similar amino acids are pink and light blue. The conserved D, D, D, QXXRW motifs are labelled. The black horizontal line marked the Cellulose_synt domain. The red box is marked with the *spo1* mutation site. The accession numbers of the CSLDs used in this study are AtCSLD1 (NP_001324935.1), AtCSLD2 (NP_197193.1), AtCSLD3 (NP_186955.1), AtCSLD4 (NP_195532.1), AtCSLD5 (NP_171773.1), AtCSLD6 (NP_174497.1), OsCSLD1 (XP_015614304.1), OsCSLD2 (XP_015643356.1), OsCSLD3 (XP_015649263.1), SPO1/OsCSLD4 (Os12g0555600), OsCSLD5 (XP_015642593.1), BdCSLD1 (XP_024317796.1), BdCSLD2 (XP_003557145.1), BdCSLD3 (XP_003573792.1), BdCSLD4 (XP_003575482.1), BdCSLD5 (XP_003568676.1), ZmCSLD1 (GRMZM2G015886), ZmCSLD2 (GRMZM5G870176), ZmCSLD3 (GRMZM2G061764), ZmCSLD4 (GRMZM2G044269), ZmCSLD5 (GRMZM2G436299).

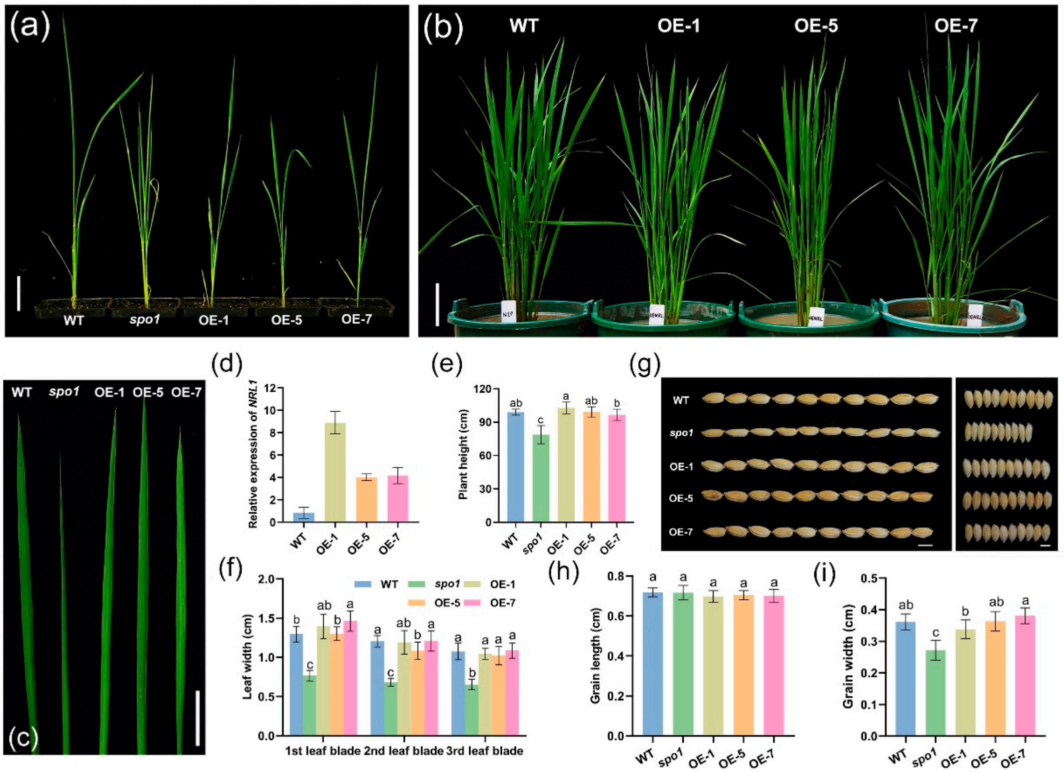

Figure S3. Characterization of overexpression (OE) plant morphology. (a) The seedlings of WT, *spo1*, OE-1, OE-5 and OE-7 plant. (b) Gross morphology of WT, OE-1, OE-5 and OE-7 plants at the tillering stage. (c) Morphology of the second leaf blades from top of WT, *spo1*, OE-1, OE-5 and OE-7 plants. (d) qRT-PCR analysis of the expression of *SPO1* in *spo1*. (e, f) Statistical analysis of plant height (e), the top three leaf blades width (f) among WT, *spo1*, OE-1, OE-5 and OE-7 plant at the mature stage. (g) Paddy rice grains of WT, *spo1*, OE-1, OE-5 and OE-7 plant. (h, i) Statistical analysis of seed length (h), seed width (i). Data presented are means ± SD: different letters indicate significant differences according to Duncan's multiple range test (5% α). Scale bars: (a) 5 cm; (b) 20 cm; (c) 5 cm; (d) 0.45cm (left) and 0.25cm (right).

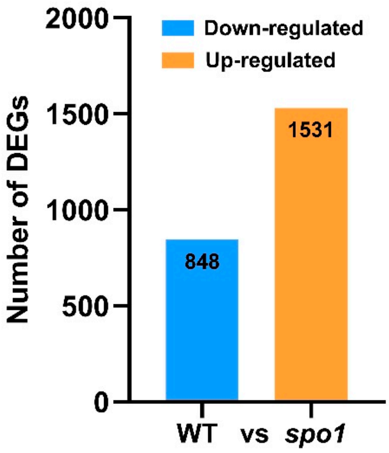

Figure S4. RNA-seq detected 2379 differentially expressed genes, of which 848 genes were downregulated, 1531 genes were significantly upregulated, fold change > 2, FDR < 0.05.

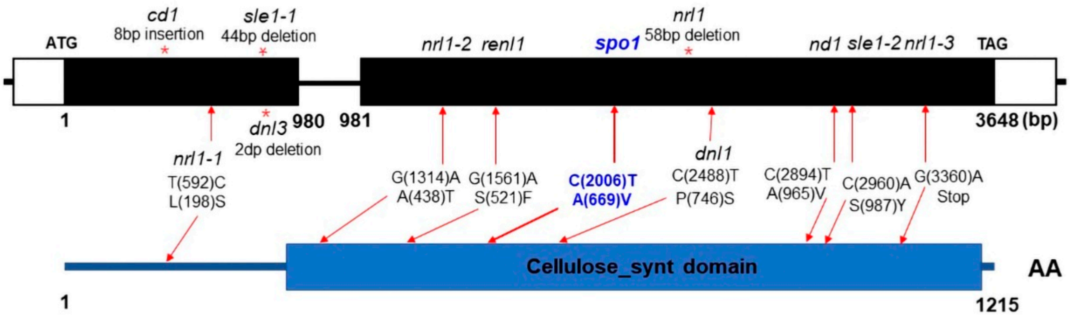

Figure S5. Comparative analysis of identified *OsCSLD4* allele mutation sites. The *OsCSLD4* gene structure and protein domain are shown above and below, respectively. The black box, black line, white box, and blue box represent exon, intron, UTR, and protein domain, respectively. Italics indicate *OsCSLD4* mutant alleles in previously studies. The blue font is a *spo1* mutant for this study. Asterisks represent the bases deletion or insertion and arrows represent single base substitution.

Table S1. The analysis of the potentially cis-elements in the promoter sequence

Motif	Distance	Sequence	Function
ABRE	-1215(+)<br>-312(-)	CACGTG<br>ACGTG	cis-acting element involved in the abscisic acid responsiveness
CGTCA-motif	-315(+)	CGTCA	cis-acting regulatory element involved in the MeJA responsiveness
TGACG-motif	-1392(-)<br>-311(-)	TGACG<br>TGACG	cis-acting regulatory element involved in the MeJA responsiveness
LTR	-63(-)	CCGAAA	cis-acting element involved in low-temperature responsiveness
MBS	-1072(-)	CAACTG	MYB binding site involved in drought inducibility
TATC-box	-1647(+)	TATCCCA	cis-acting element involved in gibberellin responsiveness
TGA-element	-1496(+)	AACGAC	auxin-responsive element
MSA-like	-478(+)	CCAAACGGCT<br>ACTTCCATCT<br>TCCACCC	cis-acting element involved in cell cycle regulation

Table S2. Summary of the primers used in this study.

Primer name	Forward primer (5'→3')	Reverse primer (5'→3')	Purpose
R12M10	ATCATTTCAGCCTGTGCC	AGCTTAATAGGGGGGACG	Gene mapping
RM101	GTGAATGGTCAAGTGACTTAGGTGGC	ACACAACATGTTCCCTCCCATGC	
RM277	CGGTCAAATCATCACCTGAC	CAAGGCTTGCAAGGGAAG	
RM17	TGCCCTGTTATTTTCTTCTCTC	GGTGATCCTTTCCCATTTCA	
RM519	AGAGAGCCCCTAAATTTCCG	AGGTACGCTCACCTGTGGAC	
RM463	TTCCCCTCCTTTTATGGTGC	TGTTCTCCTCAGTCACTGCG	
RM28433	AATAGCTGCATATACCCGGTTGG	TGTGTCTCTGATGATCCGTTTCG	
RM28449	CACCCATTGATGTGAAACTCTGG	GGATTCATGATACAGTGTGCAACG	
RM28460	ACCGCACCACGTACCTCTGG	TGCACAGCCCTATATAGCAACTCC	
RM28465	GCCTCGTAGAGACTCAAACTAAACG	GTAGCTAGCCACCAATAGCATGG	
RM3726	TACACCCACCCACATACGTCAGC	GTCGTACTCCCGGATCTTCTTCC	
RM6410	GGAAGAAGCATCTGCAGTAGTAGACG	GAATTAGCCGACAGCGTCTTGG	
RM5479	CTCACCATAGCAATCTCCTGTGC	ACTTCGTTCACTTGCATCATGG	
RM28637	TCACATGTCATACGGCTACAGACC	CACAATCACAGTGTGTGCAAAGG	
RM6411	GTGAAGGCTGTACCATCCAACG	CGGGTATTGTCGGTGTTCAGG	
R12M43	CCGCCGAGAAGAAACAAAG	CCCAAGAACAGGATTACA	
RM235	AGAAGCTAGGGCTAACGAAC	TCACCTGGTCAGCCTCTTTC	
RM28428	AACTCCTCGACTTTCGCGATATGACC	GCGGATGATTTCTCCGGTTAAATTGG	
RM6217	CGCAGATGGAGATTCTTGAAGG	ACAGCAGCAAGAGCAAGAAATCC	
RM28457	TTAGCTAGCCACTACCAAGAGAGG	GTCCTTTCGACAGTACATCTGG	
RM28451	TGAGTTCGAGTTCGATTTCTCG	TCCTTCAAGAATCTCCATCTGC	
RM28456	TGTACACTCACCTTCGCTTCTCC	CCTCTCTTCGTTCTGGAGTCTGG	
RM28621	GTGTCAACTGTCAACAAACACC	AAGGTCAGGGTTACATGATAGG	
RM28610	GAGTAAGTTCCTGCTTCTGTTTGC	GTCGAAGCTTTCATCTCTATCTCC	

Pun-*SPO1*	CGGGATCCATGTCGCGGCGGCTGTCGT	CGAGCTCCTATGGGAAGCTGAATCCGC	Transformation construct
P35s-*SPO1*-GFP	GCTCTAGAATGTCGCGGCGGCTGTCGT	CGGGATCCTGGGAAGCTGAATCCGCCG	
q*OsActin1*	GTGGTCGCCCCTCCTGAAAG	GGCTTAGCATTCTTGGGTCCG	qRT-PCR
q*SPO1*	CAGCGTCTCCCAAGCCATTC	CCACCGACATCGCCCACAG	
q*NAL1*	ACATTGGGGATGTCAAGGTTAT	ACATTGGGGATGTCAAGGTTAT	
q*NAL7*	ATCGTAGAGTTCTTCACCAT	CCAGATAAGCAGGCAGTA	
q*NRL2*	TCCCTTTCTTTTGATGAGGA	GCTACATGTAACGCCGATTC	
q*ACL1*	CTGAAGCTGAACCTCTCGCTG	GGAGCATGACGTAGATGAAGCAG	
q*ACL2*	CTCGTGCCTGTCGTCGGAG	TCGGCGAGCATGACGTAGAG	
q*RL14*	GAGGATGAACTACTATCCACCG	GTGCTCAACACTTCTGAACTTT	
q*OsZHD1*	CAGGAGCAGAAGGACAAGATG	CGACGATGAGATCAAATGCATT	
q*NAL9*	TTTATGATTGCATGTCCTGGAT	TCTCACCGCCAGCAAGTAT	
q*SLL1*	CAGCTCGCAGGTGTCCAA	CCTCCCTAGAGTGAACTCGAGACT	
q*Roc5*	CGCAAGAGGAAGAAGCGATAC	GCTCCAGTTGCGTCTTCATC	
q*RL9*	ATTCTTGCAACATGGACGCC	CATTAGCCTCTGTGATTGCC	
